# Combining LDL-C and HDL-C to predict survival in late life: The InChianti study

**DOI:** 10.1371/journal.pone.0185307

**Published:** 2017-09-28

**Authors:** Giovanni Zuliani, Stefano Volpato, Marco Dugo, Giovanni B. Vigna, Mario Luca Morieri, Marcello Maggio, Antonio Cherubini, Stefania Bandinelli, Jack M. Guralnik, Luigi Ferrucci

**Affiliations:** 1 Department of Morphology, Surgery, and Experimental Medicine, Section of Internal and Cardiopulmonary Medicine, University of Ferrara, Ferrara, Italy; 2 Department of Clinical and Experimental Medicine, Section of Geriatrics, University of Parma, Parma, Italy; 3 Institute of Gerontology and Geriatrics, INNRCA, Ancona, Italy; 4 Azienda Sanitaria Firenze, Italy; 5 Department of Epidemiology and Public Health, University of Maryland School of Medicine, Baltimore, Maryland, United States of America; 6 Longitudinal Studies Section, Clinical Research Branch, National Institute on Aging, NIH, Baltimore, Maryland, United States of America; Universita degli Studi di Napoli Federico II, ITALY

## Abstract

**Background:**

While the relationship between total cholesterol (TC) and cardiovascular disease (CVD) progressively weakens with aging, several studies have shown that low TC is associated with increased mortality in older individuals. However, the possible additive/synergic contribution of the two most important cholesterol rich fractions (LDL-C and HDL-C) to mortality risk has not been previously investigated. Our study aimed to investigate the relationship between baseline LDL-C and HDL-C, both separately and combined, and 9-years mortality in a sample of community dwelling older individuals from the InCHIANTI study.

**Methods and findings:**

1044 individuals over 64 years were included. CVD and cancer mortality were defined by ICD-9 codes 390–459 and 140–239, respectively. LDL-C <130 mg/dL (3.36 mmol/L) was defined as “optimal/near optimal”. Low HDL-C was defined as <40/50 mg/dL (1.03/1.29 mmol/L) in males/females, respectively. Nine-years mortality risk was calculated by multivariate Cox proportional hazards model. We found that, compared to subjects with high LDL-C and normal HDL-C (reference group), total mortality was significantly increased in subjects with optimal/near optimal LDL-C and low HDL-C (H.R.:1.58; 95%CI:1.11–2.25). As regards the specific cause of death, CVD mortality was not affected by LDL-C/HDL-C levels, while cancer mortality was significantly increased in all subjects with optimal/near optimal LDL-C (with normal HDL-C: H.R.: 2.49; with low HDL-C: H.R.: 4.52). Results were unchanged after exclusion of the first three years of follow-up, and of subjects with low TC (<160 g/dL—4.13 mmol/L).

**Conclusions:**

Our findings suggest that, in community dwelling older individuals, the combined presence of optimal/near optimal LDL-C and low HDL-C represents a marker of increased future mortality.

## Introduction

Cardiovascular disease (CVD) is the first cause of death in Western Countries, and its prevention is one of the main targets of health care systems. Dyslipidemia represents an important risk factor for CVD; indeed, it has been extensively demonstrated that high levels of low-density lipoprotein cholesterol (LDL-C) or apo B, as well as low levels of high-density lipoprotein cholesterol (HDL-C) or apo A-I, are associated with increased CVD risk [1–5], also in the elderly [6]. However, functional that describes death risk associated with cholesterol levels is not linear, especially in older populations. In particular, in frail individuals, low levels of total cholesterol (TC) (<160 g/dL– 4.13 mmol/L) are associated with higher mortality risk, possibly because of increase mortality due to cancer, respiratory/digestive disease, and injuries [1,7]. The relationship between TC and CVD weakens considerably with aging [6,8]. Longitudinal studies have clearly shown that, in individuals over 65 years of age, low TC is associated with increased mortality and disability [8, 9–12]. In a recent meta-analysis conducted on 19 studies, LDL-C levels were not or were inversely associated with overall mortality in older individuals [13]. On the other hand, HDLs seem to maintain their “protective” effect on CVD also in advanced age [14]; indeed, high HDL-C has been associated with survival [15], longevity [16,17], “successful” aging [18], and absence of disability [19]. To be noted, only a very few studies considered the independent and joined effect of LDL-C and HDL-C on adverse outcomes in older- adults [20,21]; moreover, the possible additive/synergic contribution of these lipoprotein fractions (which have a very different physiological role in lipid metabolism) to mortality risk has not been previously investigated. We hypothesized that both these lipoprotein might contribute to increase mortality rates in elderly people. To test this hypothesis, we evaluated the relationship between baseline LDL-C and HDL-C levels, both separately and combined, and 9-years overall, cardiovascular, and cancer mortality in a sample of community dwelling individuals enrolled into the InCHIANTI study.

## Materials and methods

This study is part of the “Invecchiare in Chianti” (Aging in the Chianti area, InCHIANTI) study, a prospective population-based study of older persons, designed by the Laboratory of Clinical Epidemiology of the Italian National Research Council of Aging (INRCA, Florence, Italy). The study included participants randomly selected from the residents in two towns of the Chianti area. A detailed description of sampling procedure and data collection method has been previously published [22]. Briefly, in 1998, 1270 persons ≥65 years were randomly selected from the population registries. Additional subjects (n = 29) were randomly selected to obtain a sample of 30 men and 30 women aged ≥ 90 years old. Of the initial 1299 subjects, 39 were not eligible. Clinical visits and assessments were performed in the study clinic and were preceded by an interview conducted at the participants’ homes. Trained interviewers administered structured questionnaires on dietary intakes, household composition, social networks, economical status, education, and general information on health and functional status. The INRCA Ethical Committee ratified the entire study protocol. The analyses presented here are based on data from 1044 individuals aged over 64 in which metabolic parameters and inflammatory mediators had been measured at baseline visit.

### Clinical chemistry parameters

All parameters were measured on fresh serum or plasma drawn after 12 h overnight fasting, after the patient has been sedentary in sitting or supine position for 15 min. Commercial enzymatic tests (Roche Diagnostics) were used for determining serum total cholesterol, triglycerides, and HDL-C concentrations. Low-density lipoprotein-cholesterol (LDL-C) was calculated by the Friedewald’s formula as follows: LDL-C: TC—(TG/5)—HDL-C. Fasting glucose, high sensitivity C reactive protein (hs-CRP), and interleukin-6 (IL-6) were measured as previously described [23]. LDL-C was dichotomized in: “optimal/near optimal”, when its value was <130 mg/dL (<3.36 mmol/L) or “high” when ≥ 130 mg/dl. (http://www.nhlbi.nih.gov/health/resources/heart/heart-cholesterol-hbc-what-html#numbers). HDL-C was considered “low” when <40mg/dL (1.03 mmol/L) in men or <50mg/mL (<1.29 mmol/L) in women (or the subjects was treated for low HDL-C) (NCEP-ATPIII-AHA/NHLBI statement of 2005) [24]. Subjects were divided into four groups based on baseline LDL-C and HDL-C:

- GROUP 1: high LDL-C and normal HDL-C levels (Reference group)
- GROUP 2: high LDL-C and low HDL-C levels
- GROUP 3: optimal/near optimal LDL-C and normal HDL-C levels
- GROUP 4: optimal/near optimal LDL-C and low HDL-C levels

### Other covariates

Weight and height were measured by using standard techniques. Body mass index (BMI) was calculated as weight (kg) divided by the square of height (m). The presence of specific medical conditions was established using standardized criteria combining self-reported history, medical records, and clinical examination. The following diseases were considered: type 2 diabetes, coronary heart disease, stroke, diagnosis of known cancer and dementia.

### Mortality follow-up

Participants were evaluated for the 3-year (2001 to 2003), 6-year (2004 to 2006) and 9-year follow-up visits (2007 to 2009). Mortality data of the original InCHIANTI cohort were collected using data from the Mortality General Registry maintained by the Tuscany Region, and the death certificates that are deposited immediately after death at the Registry office of the municipality of residence. Cardiovascular mortality, based on underlying cause of death, was defined as any cardiovascular mortality coded by the International Classification of Diseases, 9th Revision (ICD-9) by codes 390 to 459. Cancer mortality, based on underlying cause of death, was defined as any mortality related to known cancer, and coded by the ICD-9 codes 140 to 239.

### Statistical analysis

Continuous variables were expressed as mean (SD) or median (interquartile range) when necessary. Means were compared by ANOVA with Bonferroni post-hoc test for multiple comparison, while medians were compared by Mann-Whitney test. Correlations between continuous variables were tested by Pearson’s correlation. Proportions were compared by the χ^2^ test. Hazard Ratios (H.R.) for all-cause, CVD, and cancer mortality, according to LDL-C/HDL-C groups, were estimated by Cox proportional hazard regression analysis. Group 1 (high LDL-C/high HDL-C) was considered as the reference category. The assumption of proportionality of all variables introduced in the models was assessed through the analysis of Schoenfeld residuals.

The Cox models were adjusted for potential confounding factors including: age, gender, statin therapy, years of school, smoking, alcohol consumption, BMI, creatinine, uric acid, interleukin 6 (IL-6) plasma levels, serum albumin, hypertension, diabetes, Coronary Heart Disease (CHD), stroke, congestive heart failure (CHF, weight loss >4.5 kg in the last year, and diagnosis of cancer.

Analyses were performed by SPSS for Windows statistical package, version 13.0)<<?Q10>>.

## Results

The principal characteristics of the sample according to combined levels of LDL-C and HDL-C are reported in Table 1.

**Table 1:** Principal characteristics of 1044 community dwelling older individuals enrolled into the INCHIANTI study according to combined levels of LDL-C and HDL-C (LDL-C cut off: 130 mg/dl; HDL-C cut off: 40 mg/dl males, 50 mg/dl females).

	GROUP 1 (n = 470)	GROUP 2 (n = 117)	GROUP 3 (n = 331)	GROUP 4 (n = 126)	P value
	High LDL-C	High LDL-C	Optimal/near optimal LDL-C	Optimal/near optimal LDL-C	
	Normal HDL-C	Low HDL-C	Normal HDL-C	Low HDL-C	
**Male gender**	213 (44.8)	22 (19.1)°	170 (51.6)*	52 (41.9)*	**<0.001**
**Age (years)**	73.7±6.9	75.3±6.7	75.8±7.6°	77.5±8.5°	**<0.001**
**School (years)**	5.8±3.6	4.7±2.8°	5.1±3.2°	4.6±2.8°	**<0.001**
**MMSE**	26 [24; 28]	25 [23; 28]	26 [23;28]	24 [21;27]°^	**<0.001**
**Disability in BADLs**	22 (4.6)	12 (10.4)	34 (10.3)°	23 (18.6)°	**<0.001**
**Smoking habit**					
** - Never**	270 (56.8)	85 (73.9)	185 (56.2)	76 (62.9)	**0.011**
** - Previous**	135 (28.4)	15 (13.0)	101 (30.7)	31 (25.0)	
** - Present**	70 (14.8)	15 (13.1)	43 (13.1)	15 (12.1)	
**9-year Total Mortality (n/%)**	118 (25.1)	39 (33.3)	130 (39.2)°	63 (50)°*	**<0.001**
**Hypertension (n/%)**	288 (60.6)	87 (75.7)°	186 (56.5)*	79 (63.7)	**0.004**
**Diabetes (n/%)**	50 (10.5)	16 (13.9)	42 (12.8)	26 (21.0)°	**0.021**
**CHD (n/%)**	37 (7.8)	6 (5.2)	23 (7.0)	14 (11.3)	0.321
**Stroke (n/%)**	27 (5.7)	10 (8.7)	13 (4.0)	8 (6.5)	0.264
**CHF (n/%)**	15 (3.2)	7 (6.1)	19 (5.8)	17 (13.7)°	<0.001
**Cancer (n/%)**	22 (4.6)	12 (10.4)	20 (6.1)	9 (7.3)	0.117
**BMI (kg/m**^**2**^**)**	27±3.7	29±4.8°	27±4.0*	28±4.5^	**<0.001**
**Hb (g/dl)**	13.9±1.3	13.5±1.3°	13.7±1.5	13.3±1.6°	**<0.001**
**Creatinine (mg/dl)**	0.93±0.19	0.88±0.39	0.93±0.18°	1.00±0.34*^	**<0.001**
**Uric acid (mg/dl)**	5.0±1.3	5.3±1.3	5.1±1.4	6.0±2.1°*^	**<0.001**
**Albumin (g/dl)**	5.9±3.1	5.8±3.8	5.9±3.7	5.7±4.6°^	**0.009**
**Tot. Cholesterol (mg/dl)**	245±29.8	232±27.1°	188±22.8°*	176 ±30°*^	**<0.001**
**Triglycerides (mg/dl)**	113	155	87	142	**<0.001**
	[89; 145]	[124;199]°	[70; 116]°*	[101;212] °^	
**LDL-C (mg/dl)**	160±24	156±21	107±18°*	103±20°*	**<0.01**
**HDL-C (mg/dl)**	59±12	42±5°	62±15*	38±7°*^	**<0.01**
**LDL-C/HDL-C ratio**	2.79±0.63	3.80±0.80°	1.8±0.50°*	2.76±0.64*^	**<0.01**
**Glucose (mg/dl)**	88 [82; 98]	90 [82; 103]	88 [80; 99]	90 [82; 107]	0.169
**Hs-CRP (mg/L)**	2.82	3.5	2.22	4.18	**<0.001**
	[1.32; 5.49]	[1.54; 8.23]°	[1.10; 4.56]*	[1.94; 9.10]°^	
**Interleukin-6 (ng/ml)**	1.37	1.54	1.39	2.02°	**<0.001**
	[0.38; 1.37]	[0.74.; 1.34]	[0.84; 2.13]	[1.29; 3.85]	
**Hypolipemic drugs**	20 (4.2)	5 (4.3)	15 (4.5)	7 (5.6)	0.937

° VS 1 all p < 0.007;

* VS 2 all p < 0.001;

^ VS 3 all p < 0.005

In general, subjects in group 3 and 4 were older and more disabled compared with group 1, while smoking habit was less frequent in group 2. Education was higher in group 1, while the cognitive performance (Mini Mental Test Examination score) was lower in group 4 compared with group 1 and 3. Hypertension was more frequent in group 2, while diabetes and CHF were more frequent in group 4. No differences in CHD, stroke, and cancer prevalence was observed. BMI was higher while hemoglobin was lower in groups 2 and 4 compared with groups 1 and 3. Hs.CRP, IL-6, uric acid, and serum creatinine were higher, while albumin was lower in group 4. As regards plasma lipids, a trend toward a significant reduction in TC levels was observed from group 1 to 4. As expected, triglycerides levels were higher in groups 2 and 4 (low HDL-C). No differences in the prevalence of individuals taking hypolipemic drugs was observed.

### LDL-C and HDL-C as predictors of nine-year mortality

Independent of age and gender, and compared to normal HDL-C, low HDL-C (<40mg/dL (1.03 mmol/L) in men or <50mg/mL (1.29 mmol/L) in women) was associated with increased total mortality risk (Hazard Ratio:1.49; 95% Confidence Interval:1.19–1.86). Compared to high LDL-C, optimal/near optimal LDL-C (<130 mg/dL: 3.36 mmol/L)) was associated with an increased risk of death (H.R.:1.26; 95%CI:1.02–1.54, age and sex adjusted).

Next, we evaluated the overall mortality risk according to the combined levels of LDL-C and HDL-C. After 9 years, 350 subjects (33.5%) had died; in particular, 24.8% had died in group 1 (high LDL-C, normal HDL-C: 33/1000/person-year), 33.9% in group 2 (high LDL-C, low HDL-C: 46.4/100/person-year), 39.5% in group 3 (optimal/near optimal LDL-C, normal HDL-C: 58/1000/person-year), 50.8% in group 4 (optimal/near optimal LDL-C, low HDL-C: 83.1/1000/person-year)(Pearson χ^2^:37.43; p:0.0001).

**Fig 1:**
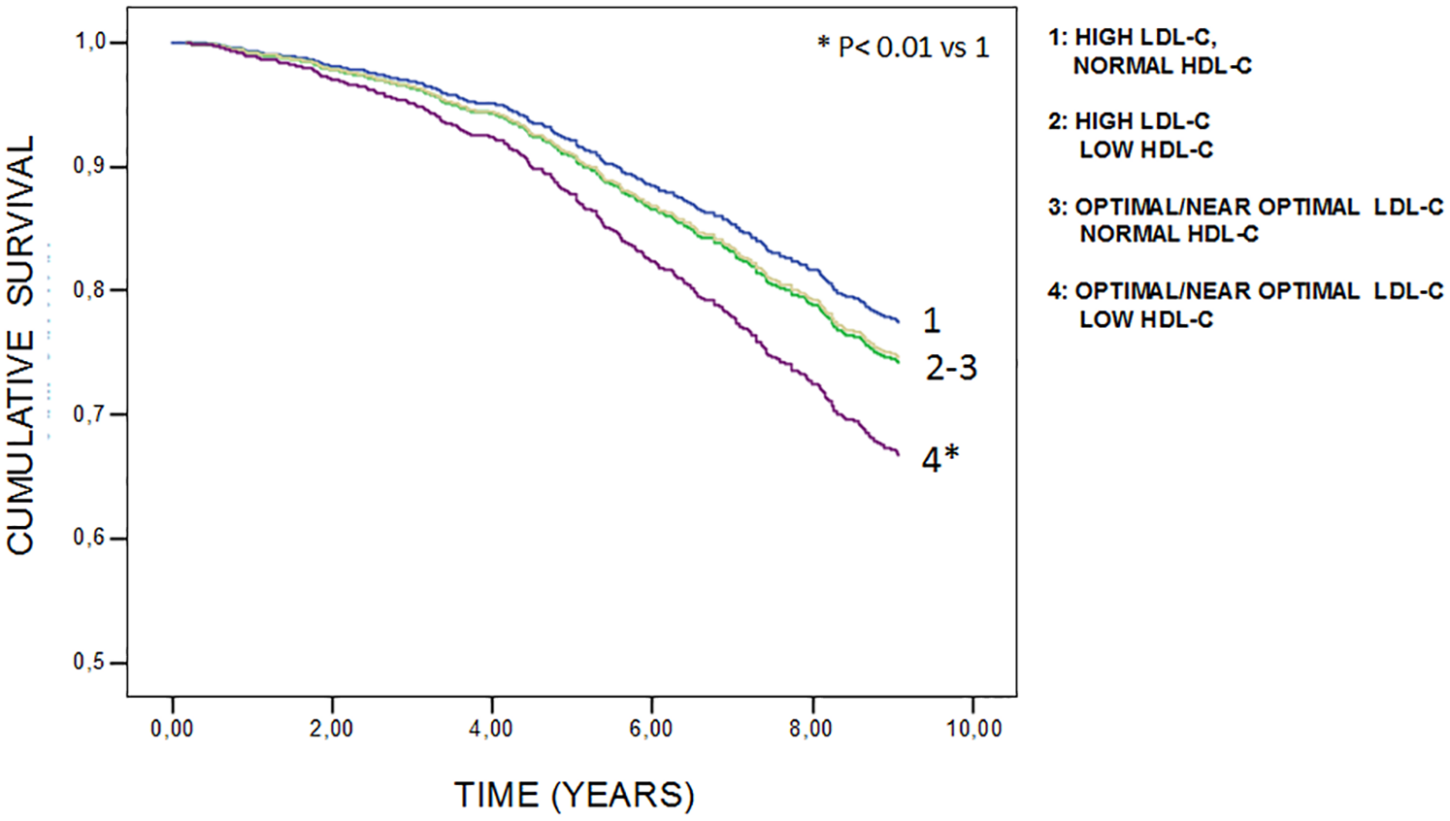
9 years cumulative survival curves by multivariate Cox proportional hazards model, in 1044 community dwelling older subjects, according to combined levels of plasma LDL-C and HDL-C (adjusted for age, gender, statin therapy, years of school, smoking, alcohol consumption, BMI, creatinine, uric acid, IL-6 plasma levels, serum albumin, hypertension, diabetes,CHD, stroke, CHF, weight loss >4.5 kg in the last year, and diagnosis of cancer).

In Fig 1 are reported the 9 years cumulative survival curves according to Cox model. After multivariate adjustment (age, gender, statin therapy, years of school, smoking, BMI, creatinine, uric acid, IL-6 plasma levels, serum albumin, hypertension, diabetes, CHD, stroke, weight loss >4.5 kg in the last year, and diagnosis of cancer), group 4 displayed a significant increase in the risk of death (H.R.:1.58; 95%CI:1.11–2.25) compared with group 1. On the contrary, a slight not significant increase in the risk was observed in group 2 (H.R.: 1.10; 95% CI:0.74–1.64), and 3 (H.R.: 1.25; 95% CI:0.96–1.67). Results of the Cox model were unchanged after exclusion of the few individuals taking hypolipidemic drugs (n = 47; data not shown). The interaction between LDL-C and HDL-C was tested by a Cox model (age-sex adjusted), and was not significant.

### Causes of death

After 9-years follow-up, the principal causes of death were CVD (46.6%) and cancer (22.6%), followed by respiratory diseases (9.4%), digestive tract diseases (4.6%), CNS diseases (4.0%), injuries (3.7%), and other causes (9.1%). In Table 2 are reported the mortality rate (%) in the four groups according to the principal causes of death. Besides CVD and cancer, all the other causes of death were collapsed into a single group (“other”) due to the small number of events.

**Table 2:** Principal causes of death after 9 year follow-up period in 1044 community dwelling older individuals enrolled into the INCHIANTI study, according to combined levels of LDL-C and HDL-C.

	Principal Causes of Death (n, % within group)			DEATHS (n, % total)
	CVD	CANCER	OTHER	
**GROUP 1 (n = 470)**	65 (55.1)	20 (16.9)	33 (28)	118 (24.8)
**High LDL-C**				
**Normal HDL-C**				
**GROUP 2 (n = 117)**	23 (59)	8 (20.5)	8 (20.5)	39 (33.9)
**High LDL-C**				
**Low HDL-C**				
**GROUP 3 (n = 331)**	54 (41.6)	31 (23.8)	45 (34.6)	130 (39.5)
**Optimal/near optimal LDL-C**				
**Normal HDL-C**				
**GROUP 4 (n = 126)**	21 (33.3)	20 (31.8)	22 (34.9)	63 (50.8)
**Optimal/near optimal LDL-C**				
**Low HDL-C**				
**TOTAL**	163 (46.6)	79 (22.6)	108 (30.8)	350 (33.5)

Pearson χ2 = 14.05; p = 0.02

As regards CVD mortality, a trend toward a progressive reduction from group 2 (high LDL-C, low HDL-C = 59%), to group 1 (high LDL-C, normal HDL-C = 55.1%), to group 3 (optimal/near optimal LDL-C, normal HDL-C = 41.6%), to group 4 (optimal/near optimal LDL-C, low HDL-C = 33.3%) was observed.

Cancer associated mortality progressively increased from group 1 (16.9%), to group 2 (20.5%), to group 3 (23.8%), to group 4 (31.8%), with a near doubling of mortality from group 1 to 4 (Pearson χ^2^:14.057; p:0.02).

In Table 3 are reported the crude mortality rates and the adjusted Hazard Ratios for the principal causes of death in the four groups of subjects. For CVD and “other” causes of mortality, no association with LDL-C/HDL-C groups could be demonstrated. On the contrary, a significant increase in the risk of death due to cancer was observed in group 3 (optimal/near optimal LDL-C, normal HDL-C = H.R.: 2.49; 95% CI:1.38–4.49), and 4 (optimal/near optimal LDL-C, low HDL-C = H.R.: 4.52; 95% CI:2.30–8.86) compared with group 1. Result were substantially unchanged after exclusion of the first three years of follow-up (data not shown).

**Table 3:** Crude mortality rate and relative adjusted Hazard Ratio for 9 years principal causes of death (cardiovascular disease, cancer, and other causes) in 1044 community dwelling older individuals enrolled into the INCHIANTI study according to combined levels of LDL-C and HDL-C.

		9 YEARS MORTALITY		
		CVD	CANCER	OTHER
**GROUP 1 (n = 470)**	**Crude rate**	17.6/1000/person-year	5.3/1000/person-year	10.1/1000/person-year
**High LDL-C**				
**Normal HDL-C**	**Hazard Ratio**	1	1	1
**GROUP 2 (n = 117)**	**Crude rate**	26.7/1000/person-year	9.3/1000/person-year	10.4/1000/person-year
**High LDL-C**				
**Low HDL-C**	**Hazard Ratio**	1.08 (CI: 0.63–1.83)	1.77 (CI: 0.76–4.12)	0.64 (CI: 0.27–1.55)
**GROUP 3 (n = 331)**	**Crude rate**	24.0/1000/person-year	13.7/1000/person-year	20.3/1000/person-year
**Optimal/near optimal LDL-C**				
**Normal HDL-C**	**Hazard Ratio**	0.96 (CI: 0.65–1.41)	**2.49 (CI: 1.38–4.49)**	1.41 (CI: 0.87–2.27)
**GROUP 4 (n = 126)**	**Crude rate**	28.1/1000/person-year	26.9/1000/person-year	28.1/1000/person-year
**Optimal/near optimal LDL-C**				
**Normal HDL-C**	**Hazard Ratio**	0.75 (CI: 0.41–1.37)	**4.52 (CI: 2.30–8.86)**	1.59 (CI: 0.78–3.21)

**H.R. adjusted for**: age, gender, statin therapy, years of school, smoking, alcohol consumption, BMI, creatinine, uric acid, IL-6 plasma levels, serum albumin, hypertension, diabetes, CHD, stroke, CHF, weight loss >4.5 kg in the last year, and diagnosis of cancer.

## Discussion

We analyzed the relationship between LDL-C and HDL-C levels and 9-year mortality in community-dwelling elderly individuals from the InCHIANTI study. Indeed, a number of studies suggest that, over 65 years, the clinical significance of plasma lipids might be different from young-adult population, since not only low HDL-C [15,17–19], but also optimal/near optimal levels of LDL-C [9,11–13,20] have been associated with negative outcomes.

Compared to individuals with high LDL-C/normal HDL-C (group 1), we found that subjects with optimal/near optimal LDL-C and low HDL-C (group 4) showed a 58% increase in overall mortality; on the other hand, the presence of just optimal/near optimal LDL-C or low HDL-C levels was not associated with increased mortality. Besides confirming the negative prognostic significance of “lower” plasma lipid levels in the elderly [7,8,13] our results suggest a specific role of LDL-C and HDL-C in predicting survival in the elderly; of interest, “low” levels of both LDL and HDL cholesterol are considered as possible biomarkers of the frailty in late life.

As expected, group 4 also showed lower TC compared with other groups. Nevertheless, only 24% of subjects had a “hypocholesterolemia” (TC<160 mg/dL—4.13 mmol/L)[7]; furthermore, the absolute difference in TC between group 3 and 4 (11 mg/dL-0.28 mmol/L), although significant, is barely meaningful from the clinical perspective. These data suggest that independent of a general condition of “hypocholesterolemia”, mortality risk might be specifically associated to the presence of “lower” values of both the LDL-C and HDL-C fractions”. Indeed, after excluding subjects with TC<160, <180 or <200 mg/dL (<4.13, 4.65, or 5.15 mmol/L), a trend toward an increase in total mortality from group 1 to 4 was still present and significant (χ^2^:24.30; p:0.0001, χ^2^:13.20; p:0.004, and χ^2^:10.85; p:0.01, respectively). Actually, some important differences between group 4 and group 1 could partially explain the observed increase in mortality rate; to minimize this potential effect, we adjusted our Cox analysis for a large number of confounders (see Methods). In addition, it has to be noted that participants in group 4, except for plasma lipids levels, were absolutely similar to subjects of group 2, which instead did not display any increase in mortality rate.

We also examined in dept the causes of 9 years death. Although crude analysis showed that CVD mortality was basically higher in group 1 and 2 (high LDL-C) compared with group 3 and 4 (optimal/near optimal LDL-C), multivariate analysis demonstrated no association between CVD mortality and LDL-C/HDL-C groups. These results are in good agreement with previous findings [6,8,13], and confirm the loss of predictive power of plasma lipids as regards CVD mortality in advanced age. On the other hand, a significant trend toward an increase in cancer mortality from group 1 to group 4 was observed, and this result was unchanged after exclusion of the first three years of follow-up (data not shown). The association between low plasma lipids and cancer has been described for many years [7]. A simplify explanation is that cancer, even in a pre-clinical phase, reduce plasma lipids (reverse causation); indeed, in some studies the association disappeared/attenuated after exclusion of the first years of follow-up [25,26]. This concept has been contradicted by other studies demonstrating a strong association even after very long follow-up, thus suggesting the possibility of a cause-effect relationship [27–31]. In our study the association between lower plasma lipids and cancer persisted after exclusion of the first three years of follow-up; in addition, we found no association between decreasing LDL-C levels and increase in cancer mortality (data not shown). Thus, we could not definitely exclude a causal relationship between “lower” levels of LDL and HDL cholesterol levels and cancer mortality. The potential mechanisms linking lower lipids to cancer remain elusive; however, since the increase in cancer mortality was observed in group 3 and 4, it might be essentially related to lower (optimal/near optimal) LDL-C levels.

Several mechanisms have been proposed to explain the association between low HDL-C and cancer, including regulation of cell cycle/apoptosis [32], modulation of cytokine production [33], antioxidative function [34], and immune-modulatory role of apo A-I [35]. The association between optimal/near optimal LDL-C levels and cancer is much more difficult to explain; it has been advocated a possible up-regulation of the mevalonate pathway in peripheral tissues [36], with production of signaling proteins such as Ras and Rho. However, Mendelian randomization studies have demonstrated that genetically reduced LDL-C (due to PCSK9, ABCG8, and apo E polymorphisms) is not associated with cancer [37]. Moreover, *in vitro* studies suggest that LDL induce cell proliferation, migration, and loss of adhesion in cancer cell lines [38]; thus, one might expect that high LDL-C levels might be associated with cancer, as indeed reported by Yang et al. [36].

We have to acknowledge some important limitations of the study. First, the size of the groups and the number of events were relatively small, and we could not further stratify our population. Thus, the association between LDL-C/HDL-C and cancer mortality might be still confounded by other factors (e.g. anorexia, weight loss, liver disease, infections, anemia), although we included many confounders into multivariate analysis (residual confounding). Second, the study was conducted in an Italian sample, and the results could not be extrapolated to other populations. Third, serum LDL-C was not measured, but was calculated by the Friedewald’s formula.

We would also underline some strength of the study. First, the study was longitudinal, and the follow-up was adequately long. Second, the study enrolled community dwelling individuals, and not hospitalized or institutionalized frail elderly people. Third, the first three years of follow-up were excluded, to reduce the possible confounding effect of undiagnosed/pre-clinical diseases, obtaining identical results.

## Conclusions

In conclusion, we found that in community dwelling older individuals CVD mortality was not associated with plasma lipid levels, and having optimal/near optimal levels of LDL-C did not prolong survival. Optimal/near optimal LDL-C, when combined with low HDL-C levels, was associated with 58% increase in total mortality, and this was principally driven by a fourfold increase in cancer mortality. Our findings suggest that in community dwelling older individuals, the combined presence of “optimal/near optimal” LDL-C and low HDL-C may represent a marker of increased future mortality.
